# Real-life use of delamanid: results from the European post-authorisation safety study

**DOI:** 10.5588/ijtldopen.24.0113

**Published:** 2024-06-01

**Authors:** N. Schönfeld, L. Barkane, I. Davoliene, M. Danilovits, S. Miliauskas, F. Ader, O.M. Kon, C. Lange, A. Duvignaud, M. Heiss-Neumann, N. Hittel, N. Lazarević, I. Knebel, A. Martin, B. Eschenbach, E. van Heumen, V. George

**Affiliations:** ^1^ Helios Klinikum Emil von Behring, Berlin, Germany;; ^2^ Riga East University Hospital, Riga, Latvia;; ^3^ Vilnius University Hospital Santaros Klinikos, Vilnius, Lithuania;; ^4^ Tartu University Hospital, Tartu, Estonia;; ^5^ Department of Pulmonology, Lithuania University of Health Sciences, Kaunas, Lithuania;; ^6^ Hospices Civils de Lyon, Département des Maladies Infectieuses et Tropicales, Lyon, France;; ^7^ Imperial College Healthcare NHS Trust, St Mary's Hospital, London, UK;; ^8^ Research Center Borstel, Leibniz Lung Center, Borstel, Germany;; ^9^ Tuberculosis Unit, German Center for Infection Research (DZIF), Hamburg-Lübeck-Borstel Riems, Borstel, Germany;; ^10^ Respiratory Medicine & International Health, University Lübeck, Lübeck, Germany;; ^11^ Baylor College of Medicine and Texas Children´s Hospital, Global TB Program, Houston, TX, USA;; ^12^ Department of Infectious Diseases and Tropical Medicine, Centre Hospitalier Universitaire de Bordeaux-Groupe Hospitalier Pellegrin, Bordeaux, France;; ^13^ Asklepios Fachkliniken München-Gauting, Gauting, Germany;; ^14^ Otsuka Novel Products, Munich, Germany;; ^15^ Otsuka Pharma, Frankfurt, Germany;; ^16^ Otsuka Pharmaceutical Development and Commercialization, Princeton, NJ, USA.

**Keywords:** tuberculosis, multidrug-resistant tuberculosis, MDR-TB, PASS, safety treatment outcomes, adverse events, European Medicines Agency

## Abstract

**BACKGROUND:**

A post-authorisation safety study (PASS) on delamanid (DLM) was conducted as part of a post-approval commitment to the European Medicines Agency. The aim of this study was to evaluate the use of DLM in a real-life setting, its safety, and treatment outcomes in patients with multidrug-resistant TB (MDR-TB).

**METHODS:**

This was a prospective, multicentric, non-interventional study conducted in the European Union. MDR-TB Regimen selection and patient monitoring were conducted in accordance with existing medical practices. Data on the use of DLM, related adverse events, and treatment outcomes were collected for up to 30 months after the first DLM dose. Descriptive summary statistics were used for continuous and categorical variables.

**RESULTS:**

Out of 86 patients, one had extrapulmonary TB. Two-thirds of the patients were treated with DLM for more than 24 weeks. The most frequent adverse drug reaction to DLM was QT interval prolongation. Resistance to DLM was detected in one patient during treatment. The treatment success rate was 77%.

**CONCLUSION:**

No new safety concerns were revealed, including in patients treated with DLM for more than 24 weeks. QT interval prolongations were well managed and did not lead to any clinically significant cardiac effects. The treatment outcomes were in line with the WHO target for Europe.

## RÉSUMÉ

**CONTEXTE:**

Une étude de sécurité post-autorisation (PASS) sur le délamanide (DLM) a été menée dans le cadre d'un engagement post-approbation auprès de l'Agence européenne des médicaments. L'objectif de cette étude était d'évaluer l'utilisation du DLM dans un contexte réel, son innocuité et les résultats du traitement chez les patients atteints de TB multirésistante (MDR-TB).

**MÉTHODES:**

Il s'agissait d'une étude prospective, multicentrique et non interventionnelle menée dans l'Union européenne. La sélection du schéma thérapeutique de la MDR-TB et le suivi des patients ont été effectués conformément aux pratiques médicales existantes. Les données sur l'utilisation du DLM, les effets indésirables connexes et les résultats du traitement ont été recueillies jusqu'à 30 mois après la première dose de DLM. Des statistiques sommaires descriptives ont été utilisées pour les variables continues et catégorielles.

**RÉSULTATS:**

Sur 86 patients, un avait une TB extrapulmonaire. Les deux tiers des patients ont été traités avec du DLM pendant plus de 24 semaines. L'effet indésirable le plus fréquent du DLM était l'allongement de l'intervalle QT. Une résistance au DLM a été détectée chez un patient pendant le traitement. Le taux de réussite du traitement était de 77%.

**CONCLUSION:**

Aucun nouveau problème de sécurité n'a été révélé, y compris chez les patients traités par le DLM pendant plus de 24 semaines. Les allongements de l'intervalle QT ont été bien gérés et n'ont pas entraîné d'effets cardiaques cliniquement significatifs. Les résultats du traitement étaient conformes à l'objectif de l'OMS pour l'Europe.

TB is an infectious disease caused by *Mycobacterium tuberculosis*. In 2022, TB affected 10.6 million people globally, resulting in 1.3 million deaths.^1^ The emergence of multidrug-resistant TB (MDR-TB) presents another global challenge.^2^ According to the 2022 WHO report, 69,000 new MDR-TB cases were estimated in the European Region in 2020. Among the 30 countries reporting the highest MDR-TB burden, nine are in the WHO European Region. Despite the progress made in TB control, the treatment success rate for MDR-TB in the European Region remains lower (55.9%) than the respective regional target (75%).^3^ Delamanid (DLM), a nitrodihydroimidazo-oxazole derivative (Deltyba™ [Otsuka Pharmaceutical Co., Ltd.]), is an oral anti-TB drug developed for the treatment of pulmonary MDR-TB. The pharmacological mode of action involves inhibition of the synthesis of mycobacterial cell wall components. DLM is highly effective both in vitro and in vivo, with specific bactericidal activity against mycobacteria.^4^ During phase 2 and phase 3 clinical trials, it demonstrated a favourable benefit-risk profile in the treatment of patients with pulmonary MDR-TB.^5,6^ On 28 April 2014, DLM received a conditional marketing authorisation within the European Union (EU) for the treatment of adults with MDR-TB when an effective treatment regimen cannot otherwise be devised for reasons of resistance or tolerability. The Summary of Product Characteristics (SmPC) was updated in 2020 and 2021 to allow for paediatric use.^7^

A post-authorisation safety study (PASS) was conducted as a commitment to obtain Deltyba’s conditional marketing authorisation approval in the EU. The primary objective was to monitor the usage of DLM in real-life settings when prescribed for the treatment of MDR-TB. The secondary objectives were to evaluate the treatment outcomes at the end of a full MDR-TB treatment period, as defined by the WHO,^8^ and/or national guidelines, and to monitor the safety of DLM prescribed as part of an appropriate combination regimen (ACR) designed by the treating physician according to existing practices.

## METHODS

This study was designed as an EU-wide, prospective, multicentric, non-interventional, PASS in patients with MDR-TB who were prescribed DLM with an ACR. The study protocol expected the end of enrolment after including 250 patients or after the 4-year enrolment period, whichever occurred first. The estimation was based on the incidence of pulmonary MDR-TB in the EU,^9^ the DLM indication as per the SmPC effective at that time, and the dates of anticipated launch in EU countries. The follow-up period was up to 30 months after the first DLM dose. In agreement with the non-interventional nature and the primary objective of the PASS, no specific inclusion and exclusion criteria were detailed in the protocol, except in Germany. The German Health Authority requested a separate protocol specifying the label indication as inclusion and contra-indication as exclusion criteria, in line with the SmPC effective at the time. Inclusion criteria were adult patients with pulmonary MDR-TB who would use DLM as a part of an ACR in the absence of an otherwise effective treatment regimen because of resistance or tolerability. Contraindicated was use in patients concomitantly taking strong inducers of CYP3A4, patients with serum albumin <2.8 g/dL, and those with known hypersensitivity to the active substance or any of the excipients. All examinations, assessments, and treatment monitoring were performed according to existing clinical practices and the protocol did not define a schedule of visits or additional diagnostic, therapeutic, or monitoring procedures. Patient follow-up and data collection occurred at regular monthly visits or at intervals defined at the local/national level. Data records included, but were not limited to, patient’s age, sex, medical history, treatment indication, DLM dosage, method of administration (directly observed treatment [DOT] or self-administration), duration of DLM use, ACR and other concomitant medications, laboratory findings, including drug susceptibility testing (DST), electrocardiogram (ECG) and other investigations.

Adverse events (AEs) and other safety information were collected and recorded at each regular visit. The Medical Dictionary for Regulatory Activities v24.1 was used for coding AEs. As per protocol, the treating physicians classified AEs based on a three-point grading scale with intensity of either mild (discomfort, no disruption of daily activity), moderate (discomfort affects normal daily activity), or severe (inability to work/perform normal daily activity). Follow-up lasted until the reported conditions had resolved, stabilised or returned to baseline. Analysis of AEs considered the causality assessment of the treating physician. AEs were considered DLM-related if reported as ‘related’, ‘probably related’ or ‘possibly related’, while ‘unlikely related’ and ‘not related’ were considered as ‘not related’. Prolonged QT on electrocardiogram (ECG) was the safety event of utmost interest. The treatment outcome for each patient was assigned by the treating physician according to the WHO definitions and reporting framework for TB.^8^ No formal hypotheses were planned for this study. Intended statistical analyses were descriptive summary statistics, including mean, standard deviation, median, and range for continuous variables; and frequency counts, percentages (*n* [%]), and 95% confidence intervals (CIs) for categorical variables.

Submission of the DLM PASS dossier to Ethics Committees and/or National Competent Authorities was in line with the national requirements of the participating EU countries. Depending on the country-specific legislation, approval or an acknowledgement of receipt notification was obtained. Data collection started after the patient or their legal representative signed the informed consent form (ICF).

## RESULTS

### Patient disposition, demographics, and baseline characteristics

During the 4-year enrolment period (12 August 2016–28 July 2020), 88 patients with MDR-TB were included at 11 clinical sites in: Lithuania (*n* = 31), Germany (*n* = 20), Latvia (*n* = 16), Estonia (*n* = 14), France (*n* = 3), and the United Kingdom (*n* = 2). Two patients were excluded from the analysis dataset due to invalid ICF. Of the 86 patients in the analysed dataset, 85 had pulmonary MDR-TB and 1 had extrapulmonary MDR-TB. All enrolled participants were adults (age ≥18 years); the mean age was 40 years (range 18–72), and 64% were male. Most of them (72%) were previously treated for pulmonary TB (13%) or MDR-TB (59%). At baseline, MDR-TB was confirmed using solid or liquid culture media in 93% of cases. Chest X-ray at baseline revealed unilateral or bilateral cavitary disease in 58% of patients with pulmonary MDR-TB.

### Treatment exposure, compliance, and outcome

Treatment duration with DLM varied. In 14% of the participants, it lasted less than 24 weeks. Reasons included the following: withdrawn consent for participation in the study, drug resistance at baseline, loss to follow-up (LTFU), permanent discontinuation due to AE, or death. One-fifth of the patients were treated with DLM for 24 weeks and two-thirds were treated for longer than 24 weeks. The median treatment duration was 26 weeks (range: 0.3–113). During the 24 weeks of treatment, DLM administration was under DOT for 97% patients. DOT was combined with self-administration in four patients. A single participant practised self-administration only. All patients received DLM 100 mg twice a day in line with the SmPC posology during the treatment period of 24 weeks. Label posology was followed up in 75% of patients receiving DLM for >24 weeks. One patient received 200 mg once daily (QD) and 23% received DLM 100 mg QD. In all but one patient, DLM was combined with three or more anti-TB medications. The most commonly used ACR drugs were linezolid (80%), moxifloxacin (54%), and cycloserine (49%).

Treatment success, as per the WHO outcome definitions,^8^ was reported for 77% of patients: 57% were cured and 20% completed treatment (Table). DLM resistance was found in two participants. One had a DLM-resistant strain at baseline. Previous DLM use was not reported. As per the DST results, DLM was discontinued, and the patient completed treatment with other anti-TB medications. In another patient with cavitary extensively drug-resistant TB and no baseline DLM DST, DLM resistance was detected after 15 months of unsuccessful treatment. Due to the extensive drug resistance profile of the strain, the patient underwent pneumectomy and continued taking DLM and ACR. ‘Treatment completed’ outcome was reported 6 months after surgery.

**Table.** Frequency and percentage of final treatment outcome (enrolled set).

Final treatment outcome	Delamanid (*n* = 86)	
	*n* (%)	95% CI*
Cured^†^	49 (57.0)	46.5–67.4
Treatment completed^‡^	17 (19.8)	11.4–28.2
Lost to follow-up	11 (12.8)	5.7–19.8
Treatment failed^§^	1 (1.2)	0–6.31
Death	3 (3.5)	0–7.4
Not evaluated^¶^	5 (5.8)	1.9–13.0

* The 95% confidence interval is based on a binomial distribution (for example cured vs. not cured) using the PROC FREQ procedure (SAS Institute, Cary, NC, USA).

† Patients who completed the treatment as recommended by the national policy without evidence of failure and three or more consecutive cultures taken at least 30 days apart were negative after the intense phase.

‡ Patients who completed the treatment as recommended by the national policy without evidence of failure, but no record that three or more consecutive cultures taken at least 30 days apart were negative after the intensive phase.

§ Patient for whom treatment was terminated or there was a need for permanent regimen change of at least two anti-TB drugs.

¶ A patient for whom no treatment outcome was assigned (e.g., transferred out cases). CI = confidence interval.

### Safety

One or more AEs were reported in 92% patients. At least one serious adverse event (SAE) was registered in 24% of patients, 19% had severe AEs, 6% had AEs that led to permanent DLM discontinuation, and 4% of patients died. The most frequent AEs assessed causally related to DLM were prolonged QT on ECG (10.5%), nausea (7.0%), and vomiting (3.5%). AEs that occurred after the first dose of DLM in more than 3% of enrolled participants with their incidence, mean frequency, causality to DLM, severity, seriousness, and DLM discontinuation are presented in Supplementary Data Table S1.

The treating physicians assessed the causality of the three fatal outcomes that were not related to DLM. A severely immunocompromised patient with TB-HIV co-infection died 8 months after the initiation of DLM and ACR. Non-adherence to antiretroviral and anti-TB treatment and progression of MDR-TB/HIV with *Pneumocystis jirovecii* and *Cytomegalovirus pneumonia* led to death. Another patient who completed 24 weeks of DLM treatment died 6 months later in the continuation phase of the treatment: a potentially insufficient anti-TB treatment regimen in combination with underlying chronic hepatitis C contributed to the worsening of MDR-TB. The third patient, with a history of chronic obstructive pulmonary disease, died of an acute myocardial infarction 2 months after starting treatment with DLM and ACR. Coronary angiogram showed 95% stenosis of the S5 segment, and angioplasty did not reverse the patient’s cardiogenic shock. Apart from one patient who permanently discontinued DLM due to baseline DST DLM resistance, 5% of patients permanently discontinued DLM due to AE: 1 due to a moderate ECG QT prolongation, 1 patient with hepatic cytolysis, 1 with mild tinnitus, and 1 with mild events of chills, eye pain, and moderate nausea.^1^

ECG QT prolongation and cardiac, hepatobiliary, and psychiatric disorders, as AEs of special interest, are presented in Supplementary Data Table S2. For all patients, ECG recordings were documented at baseline. Due to a non-interventional study design, the frequency of ECG monitoring was not prescribed by the protocol. The mean values of QTcB and QTcF remained overall constant during the study. Ten patients with QT prolongations were recorded within the range of 11–373 days (median: 180) from DLM initiation. All patients concomitantly received a fluoroquinolone, and most were additionally treated with bedaquiline and/or clofazimine (CFZ). None of the QT prolongations was considered severe by the treating physicians, although three patients had QTc >500 ms. In response to QT prolongations, DLM use was permanently discontinued in a single patient with QTc = 490 ms (treated concomitantly with bedaquiline, CFZ and levofloxacin) and temporarily discontinued in three patients with QTc > 500 ms (receiving additionally CFZ and/or moxifloxacin: one was assessed to not be causally related to DLM due to the history of alcoholic hepatitis and chronic pancreatitis with diarrhoea and consecutive hypokalaemia). No electrolyte or albumin disturbances were reported with QT prolongations. One QTc prolongation of >500 ms did not recover 3 months after temporary discontinuation and restart of DLM, whereas the patient continuously received CFZ. There were nine hepatic AEs reported in eight patients, all with the outcome recovered. In two patients, one with hepatic cytolysis and one with acute hepatitis, causality was assessed as related to DLM and other ACR medications; one had a history of chronic hepatitis C. Other hepatic AEs were not attributed to DLM, including two cases of toxic hepatitis confounded by concomitant medications and a history of alcohol use in one of the patients. Nearly a quarter of patients experienced psychiatric disorders, mostly sleep-related (insomnia/sleep disorder, 13%). Typically, the ACR included 1–2 anti-TB drugs with potential psychiatric effects: cycloserine, terizidone, and/or fluoroquinolones. Overall, two non-serious psychiatric AEs, an event of moderate depression and an event of severe anxiety, were attributed to DLM. The potentially clinically relevant haematological findings were decreased haemoglobin (12 patients), leukocytosis (*n* = 6), leukopenia (*n* = 3), and thrombocytopenia (*n* = 2) confounded by underlying TB and concomitant use of linezolid.

## DISCUSSION

Assessment of real-life practises within the DLM EU PASS showed that the treating physicians followed the SmPC recommendations. Adult MDR-TB patients with limited therapeutic options were included, all but one with pulmonary disease. Most patients had a history of MDR-TB treatment and presented with lung cavitations at baseline. DLM was typically combined with three or more anti-TB drugs. DOT was implemented at all sites during the initial/inpatient phase of treatment and largely in the treatment continuation phase. In two-thirds of PASS participants, the treating physicians opted for extended use of DLM until the end of the MDR-TB treatment, deviating from the SmPC recommended 6-month administration. This was likely due to the need for effective and tolerable anti-TB treatments for MDR-TB. Overall, the AEs, laboratory evaluations, ECGs, and other findings in this study are in line with the DLM clinical trial and post-marketing data collected from all sources. Prolonged ECG QT interval and gastrointestinal complaints were the most frequently reported AEs attributed to the use of DLM. QT prolongations, occurring in patients who concomitantly used 2–4 anti-TB drugs with QT prolonging potential, rarely showed values over 500 ms. The risk of QT prolongation was effectively managed via ECG monitoring, and no clinically significant cardiac events were reported. The incidence and severity are comparable with the results from several other studies, systematic reviews, and meta-analyses reporting DLM use and its combination with QT-prolonging anti-TB agents.^10–16^ No new safety risks for DLM have been identified in this study. For AEs assessed as related to DLM, the role of confounders, such as concomitant medications and concurrent medical conditions, should be considered. The safety profile in patients on extended DLM use did not differ from that observed during the initial 24 weeks of treatment. Other studies have reported similar safety, tolerability, and efficacy results following DLM extended use.^10,12,17–23^

Although the study recruited difficult-to-treat MDR-TB patients who received DLM due to limited or no remaining treatment options, treatment success outcome (77%) was above the WHO target for Europe (75% for MDR-TB).^3^ The outcome results are comparable with results reported in other studies.^10,11,24–26^ Of note, one patient had baseline resistance to DLM, and in another patient, resistance to DLM developed during the study.

This study was planned to be EU-wide, and its main limitation is the coverage of only six countries with a significantly lower total number of enrolled participants (*n* = 88) than the anticipated 250. Coverage and enrolment were mostly impacted by a steady decline in the MDR-TB incidence in Estonia, Latvia, and Lithuania, delayed or no DLM launch in some EU countries, low recruitment potential in France and the United Kingdom, the Group C position of DLM in the 2019 WHO guidelines,^27^ and the COVID-19 pandemic. Results for DLM use in a real-life setting therefore apply to Baltic countries and Germany. Due to the limited number of included participants, the results are not generalisable to elderly or HIV-positive subpopulations. In addition, extension of the indication for DLM use to the paediatric population was approved in the EU after the enrolment had been completed; therefore, no paediatric patients were included in the study.

## CONCLUSION

The objectives of the DLM PASS (to monitor and document real-life usage of DLM in medical practice and to assess its safety and outcome data) were met. The data from this study confirmed DLM’s use in agreement with the approved indication and its favourable safety profile.

## Supplementary Material


