# Supplementary material for: Real-life use of delamanid: results from the European post-authorisation safety study
Source: IJTLD Open. 2024 Jun 1;1(6):274–8. doi: 10.5588/ijtldopen.24.0113 (PMC11249655; doi:10.5588/ijtldopen.24.0113)
Supplement: Supplementary file 1 [file ijtld_open_june_0113_suppl_data.pdf]

© 2024 The Authors http://dx.doi.org/10.5588/ijtldopen.24.0113

Real-life use of delamanid: results from the European post-authorisation safety study

Supplementary Table S1. Treatment Emergent Adverse Events That Occurred in More Than 3% of Participants (Enrolled Set)

System Organ Class/ MedDRA Preferred Term	Incidence(%)	Mean Frequency (Number of AEs)	DLM Related (%)	Severe (%)	Serious (%)	Discontinued DLM (%)
**Blood and Lymphatic System Disorders**						
Anaemia	10 (11.6 %)	14.0 % (12)	0 (0.0 %)	0 (0.0 %)	0 (0.0 %)	0 (0.0 %)
Neutropenia	5 (5.8 %)	5.8 % (5)	1 (1.2 %)	0 (0.0 %)	0 (0.0 %)	0 (0.0 %)
**Cardiac Disorders**						
Palpitations	3 (3.5 %)	3.5 % (3)	2 (2.3 %)	0 (0.0 %)	0 (0.0 %)	0 (0.0 %)
**Ear and Labyrinth Disorders**						
Hypoacusis	4 (4.7 %)	4.7 % (4)	0 (0.0 %)	0 (0.0 %)	0 (0.0 %)	0 (0.0 %)
Tinnitus	7 (8.1 %)	8.1 % (7)	1 (1.2 %)	0 (0.0 %)	0 (0.0 %)	1 (1.2 %)
**Eye Disorders**						
Optic Neuropathy	3 (3.5 %)	3.5 % (3)	1 (1.2 %)	0 (0.0 %)	1 (1.2 %)	0 (0.0 %)
**Gastrointestinal Disorders**						
Abdominal Pain Upper	4 (4.7 %)	5.8 % (5)	0 (0.0 %)	0 (0.0 %)	0 (0.0 %)	0 (0.0 %)
Diarrhoea	7 (8.1 %)	9.3 % (8)	0 (0.0 %)	0 (0.0 %)	0 (0.0 %)	0 (0.0 %)
Dyspepsia	3 (3.5 %)	3.5 % (3)	0 (0.0 %)	0 (0.0 %)	0 (0.0 %)	0 (0.0 %)
Nausea	19 (22.1 %)	26.7 % (23)	6 (7.0 %)	0 (0.0 %)	0 (0.0 %)	1 (1.2 %)
Vomiting	10 (11.6 %)	20.9 % (18)	3 (3.5 %)	0 (0.0 %)	0 (0.0 %)	0 (0.0 %)
**General Disorders and Administration Site Conditions**						
Asthenia	5 (5.8 %)	7.0 % (6)	1 (1.2 %)	0 (0.0 %)	0 (0.0 %)	0 (0.0 %)
**Infections and Infestations**						
Tuberculosis	3 (3.5 %)	3.5 % (3)	0 (0.0 %)	3 (3.5 %)	3 (3.5 %)	0 (0.0 %)
**Investigations**						
Blood Uric Acid Increased	3 (3.5 %)	3.5 % (3)	0 (0.0 %)	0 (0.0 %)	0 (0.0 %)	0 (0.0 %)
Electrocardiogram QT Prolonged	10 (11.6 %)	11.6 % (10)	9 (10.5 %)	0 (0.0 %)	0 (0.0 %)	1 (1.2 %)
**Metabolism and Nutrition Disorders**						
Decreased Appetite	6 (7.0 %)	7.0 % (6)	1 (1.2 %)	0 (0.0 %)	0 (0.0 %)	0 (0.0 %)
Folate Deficiency	4 (4.7 %)	4.7 % (4)	0 (0.0 %)	0 (0.0 %)	0 (0.0 %)	0 (0.0 %)
Hypokalaemia	5 (5.8 %)	8.1 % (7)	2 (2.3 %)	0 (0.0 %)	0 (0.0 %)	0 (0.0 %)
Vitamin D Deficiency	7 (8.1 %)	8.1 % (7)	0 (0.0 %)	1 (1.2 %)	0 (0.0 %)	0 (0.0 %)
**Musculoskeletal and Connective Tissue Disorders**						
Arthralgia	8 (9.3 %)	9.3 % (8)	0 (0.0 %)	0 (0.0 %)	0 (0.0 %)	0 (0.0 %)
**Nervous System Disorders**						
Epilepsy	5 (5.8 %)	5.8 % (5)	0 (0.0 %)	2 (2.3 %)	1 (1.2 %)	0 (0.0 %)
Headache	4 (4.7 %)	4.7 % (4)	2 (2.3 %)	0 (0.0 %)	0 (0.0 %)	0 (0.0 %)
Polyneuropathy	5 (5.8 %)	5.8 % (5)	0 (0.0 %)	0 (0.0 %)	0 (0.0 %)	0 (0.0 %)
**Psychiatric Disorders**						
Anxiety	5 (5.8 %)	5.8 % (5)	1 (1.2 %)	1 (1.2 %)	0 (0.0 %)	0 (0.0 %)
Borderline Personality Disorder	3 (3.5 %)	3.5 % (3)	0 (0.0 %)	0 (0.0 %)	0 (0.0 %)	0 (0.0 %)
Depression	3 (3.5 %)	4.7 % (4)	1 (1.2 %)	0 (0.0 %)	1 (1.2 %)	0 (0.0 %)
Insomnia	9 (10.5 %)	10.5 % (9)	0 (0.0 %)	0 (0.0 %)	0 (0.0 %)	0 (0.0 %)
**Renal and Urinary Disorders**						

Supplementary Table S1. Treatment Emergent Adverse Events That Occurred in More Than 3% of Participants (Enrolled Set)						
**System Organ Class/ MedDRA Preferred Term**	**Incidence(%)**	**Mean Frequency (Number of AEs)**	**DLM Related (%)**	**Severe (%)**	**Serious (%)**	**Discontinued DLM (%)**
Acute Kidney Injury	3 (3.5 %)	3.5 % (3)	0 (0.0 %)	0 (0.0 %)	1 (1.2 %)	0 (0.0 %)
Renal Failure	7 (8.1 %)	10.5 % (9)	1 (1.2 %)	0 (0.0 %)	1 (1.2 %)	0 (0.0 %)
**Respiratory, Thoracic and Mediastinal Disorders**						
Chronic Obstructive Pulmonary Disease	4 (4.7 %)	4.7 % (4)	0 (0.0 %)	0 (0.0 %)	0 (0.0 %)	0 (0.0 %)
Cough	4 (4.7 %)	4.7 % (4)	0 (0.0 %)	0 (0.0 %)	0 (0.0 %)	0 (0.0 %)
Dyspnoea	3 (3.5 %)	3.5 % (3)	0 (0.0 %)	0 (0.0 %)	0 (0.0 %)	0 (0.0 %)
Haemoptysis	4 (4.7 %)	4.7 % (4)	0 (0.0 %)	0 (0.0 %)	1 (1.2 %)	0 (0.0 %)
**Skin and Subcutaneous Tissue Disorders**						
Acne	4 (4.7 %)	4.7 % (4)	1 (1.2 %)	0 (0.0 %)	0 (0.0 %)	0 (0.0 %)
Dermatitis Allergic	5 (5.8 %)	5.8 % (5)	0 (0.0 %)	0 (0.0 %)	0 (0.0 %)	0 (0.0 %)
Pruritus	5 (5.8 %)	5.8 % (5)	2 (2.3 %)	0 (0.0 %)	0 (0.0 %)	0 (0.0 %)
**Vascular Disorders**						
Hypertension	4 (4.7 %)	4.7 % (4)	0 (0.0 %)	0 (0.0 %)	0 (0.0 %)	0 (0.0 %)

DLM: Delamanid

Subjects are counted once, per SOC, for the most severe of multiple occurrences of a specific system organ class.
The total number of enrolled participants (86) has been used as the denominator for all percentages.

Supplementary Table S2. Treatment Emergent Adverse Events of Special Interest (Enrolled Set)

System Organ Class/ MedDRA Preferred Term	Incidence(%)	DLM Related (%)	Severe (%)	Serious (%)	Discontinued DLM (%)
**Cardiac Disorders**					
Myocardial Infarction	1 (1.2 %)	0 (0.0 %)	1 (1.2 %)	1 (1.2 %)	0 (0.0 %)
Palpitations	3 (3.5 %)	2 (2.3 %)	0 (0.0 %)	0 (0.0 %)	0 (0.0 %)
Tachycardia	2 (2.3 %)	0 (0.0 %)	0 (0.0 %)	0 (0.0 %)	0 (0.0 %)
Wolff-Parkinson-White Syndrome	1 (1.2 %)	0 (0.0 %)	0 (0.0 %)	0 (0.0 %)	0 (0.0 %)
**Hepatobiliary Disorders**					
Hepatic Cytolysis	1 (1.2 %)	1 (1.2 %)	1 (1.2 %)	1 (1.2 %)	1 (1.2 %)
Hepatitis	1 (1.2 %)	0 (0.0 %)	1 (1.2 %)	1 (1.2 %)	0 (0.0 %)
Hepatitis Acute	1 (1.2 %)	1 (1.2 %)	0 (0.0 %)	1 (1.2 %)	0 (0.0 %)
Hepatitis Toxic	2 (2.3 %)	0 (0.0 %)	1 (1.2 %)	1 (1.2 %)	0 (0.0 %)
**Investigations**					
Alanine Aminotransferase Increased	1 (1.2 %)	0 (0.0 %)	1 (1.2 %)	0 (0.0 %)	0 (0.0 %)
Aspartate Aminotransferase Increased	1 (1.2 %)	0 (0.0 %)	1 (1.2 %)	0 (0.0 %)	0 (0.0 %)
Blood Bilirubin Increased	1 (1.2 %)	0 (0.0 %)	0 (0.0 %)	0 (0.0 %)	0 (0.0 %)
Electrocardiogram QT Prolonged	10 (11.6 %)	9 (10.5 %)	0 (0.0 %)	0 (0.0 %)	1 (1.2 %)
Transaminases Increased	1 (1.2 %)	0 (0.0 %)	0 (0.0 %)	0 (0.0 %)	0 (0.0 %)
**Psychiatric Disorders**					
Adjustment Disorder	1 (1.2 %)	0 (0.0 %)	0 (0.0 %)	0 (0.0 %)	0 (0.0 %)
Alcohol Abuse	1 (1.2 %)	0 (0.0 %)	1 (1.2 %)	0 (0.0 %)	0 (0.0 %)
Alcohol Withdrawal Syndrome	1 (1.2 %)	0 (0.0 %)	0 (0.0 %)	0 (0.0 %)	0 (0.0 %)
Anxiety	5 (5.8 %)	1 (1.2 %)	1 (1.2 %)	0 (0.0 %)	0 (0.0 %)
Anxiety Disorder	1 (1.2 %)	0 (0.0 %)	0 (0.0 %)	0 (0.0 %)	0 (0.0 %)
Borderline Personality Disorder	3 (3.5 %)	0 (0.0 %)	0 (0.0 %)	0 (0.0 %)	0 (0.0 %)
Depression	3 (3.5 %)	1 (1.2 %)	0 (0.0 %)	1 (1.2 %)	0 (0.0 %)
Generalised Anxiety Disorder	1 (1.2 %)	0 (0.0 %)	0 (0.0 %)	0 (0.0 %)	0 (0.0 %)
Hallucination	1 (1.2 %)	0 (0.0 %)	0 (0.0 %)	0 (0.0 %)	0 (0.0 %)
Insomnia	9 (10.5 %)	0 (0.0 %)	0 (0.0 %)	0 (0.0 %)	0 (0.0 %)
Panic Reaction	1 (1.2 %)	0 (0.0 %)	1 (1.2 %)	0 (0.0 %)	0 (0.0 %)
Personality Disorder	1 (1.2 %)	0 (0.0 %)	0 (0.0 %)	0 (0.0 %)	0 (0.0 %)
Schizophrenia	1 (1.2 %)	0 (0.0 %)	1 (1.2 %)	0 (0.0 %)	0 (0.0 %)
Sleep Disorder	2 (2.3 %)	0 (0.0 %)	0 (0.0 %)	0 (0.0 %)	0 (0.0 %)
Somatic Symptom Disorder	1 (1.2 %)	0 (0.0 %)	0 (0.0 %)	0 (0.0 %)	0 (0.0 %)

DLM: Delamanid

subjects are counted once, per SOC, for the most severe of multiple occurrences of a specific system organ class.
The total number of enrolled participants (86) has been used as the denominator for all percentages.
